# Access to treatment for HBV infection and its consistency with 2008 European guidelines in a multicentre cross-sectional study of HIV/HBV co-infected patients in Italy

**DOI:** 10.1186/1756-0500-6-153

**Published:** 2013-04-17

**Authors:** Giorgio Antonucci, Francesco Mazzotta, Claudio Angeletti, Enrico Girardi, Massimo Puoti, Giulio De Stefano, Paolo Grossi, Nicola Petrosillo, Gabriella Pagano, Giovanni Cassola, Anna Orani, Caterina Sagnelli, Orlando Armignacco, Evangelista Sagnelli

**Affiliations:** 1 Italian Society of Infectious & Tropical Diseases (SIMIT), Florence, Italy; 2 Department of Infectious Diseases, Santa Maria Annunziata Hospital, ASL Firenze, Florence, Italy; 3 National Institute for Infectious Diseases L. Spallanzani, Rome, Italy; 4 Institute of Infectious & Tropical Diseases, University of Brescia, Brescia, Italy; 5 Infectious Diseases Unit, Madonna delle Grazie Hospital, Matera, Italy; 6 Infectious Diseases Unit, Insubria University, Varese, Italy; 7 Infectious Diseases Unit, San Martino Hospital, Genoa, Italy; 8 Infectious Diseases Unit, Galliera Hospital, Genoa, Italy; 9 Infectious Diseases Unit, Alessandro Manzoni Hospital, Lecco, Italy; 10 Infectious Diseases Unit, Second University of Naples, Naples, Italy; 11 Infectious Diseases Unit, Belcolle Hospital, Viterbo, Italy

**Keywords:** Cohort study, Hepatitis B chronic, HIV infections/complications, Antiviral agents/therapeutic use, Health Services accessibility, Guidelines adherence

## Abstract

**Background:**

A survey was performed in 2008 to evaluate the profiles of patients with chronic hepatitis B cared for by Italian Infectious Diseases Centers (IDCs).

This analysis describes: i) factors associated with access to the anti-HBV treatment in a cohort of HIV/HBV co-infected patients cared for in tertiary centers of a developed country with comprehensive coverage under the National Health System (NHS); ii) consistency of current anti-HBV regimens with specific European guidelines in force at the time of the study and factors associated with the receipt of sub-optimal regimens.

**Methods:**

The study focuses on 374 (87.6%) treated patients at some point in their life out of the 427 tested HIV/HBV positive. It is multicentre, cross-sectional in the design. To account for missing values, a Multiple Imputation method is used.

**Results:**

Three hundred and thirty-four (89.3%) patients were currently treated. The most common current regimen was combination therapy of tenofovir (TDF) plus LAM/FTC (lamivudine/emtricitabine) (n = 235, 70.4%), as part of antiretroviral treatment.

In the multivariate analysis, an increased chance of getting treated was independently associated with increasing years since HBV diagnosis (2–10 years, p <0.001; >10 years, p <0.001).

Patients consistently treated with European AIDS Clinical Society (EACS) 2008 guidelines were 255 (76.6%), of whom 202 (79.2%) with an indication to an anti-HIV treatment, 30 (11.8%)without an indication, and 21 (8.2%) with cirrhosis. Among the 78 not-consistent patients, LAM mono-therapy (n = 60, 76.9%) was the most common regimen, 34 (56.7%) of them showing HBV DNA load below 1x10^3^ IU/mL.

Previous anti-HBV treatment (p = 0.01) and a triple HDV co-infection (p = 0.03) reduced the chance of not-consistent regimens. Conversely, HCV co-infection was independently associated with an increased odds ratio of being inconsistently treated (p = 0.004).

**Conclusion:**

Our study shows that Italian IDCs treat for HBV infection the vast majority of HIV/HBV co-infected patients with no disparities limiting access to antiviral therapy. In approximately two-thirds of the patients on treatment, anti-HBV regimens are consistent with 2008 EACS guidelines. Finally, our study identifies scenarios in which clinical practice deviates from recommendations, as in case of sub-optimal regimens with effective anti-HBV response.

## Background

Human immunodeficiency virus (HIV) and hepatitis B virus (HBV) may both result in chronic diseases, cancer, and death, representing an heavy burden for the Health Care Systems of different countries. At present, about 33 million individuals are estimated living with HIV infection and 400 million with HBV infection worldwide [1,2].

Shared ways of transmission for both viruses favour theoccurrence of HIV/HBV co-infection, whose frequency reflects the endemicity level of each single infection in different geographic areas [3]. In European countries, approximately 10% of the HIV-infected persons are concurrently co-infected with HBV [4]. Furthermore, as deaths from AIDS-related causes have declined following the introduction of combined antiretroviral therapy [cART), HBV infection has emerged as a cause of death in co-infected populations [5].

Such considerations along with new anti-HBV drugs becoming available prompted in 2008 European AIDS Clinical Society (EACS) to update guidelines for the clinical management and treatment of chronic hepatitis B (CHB) and hepatitis C virus (HCV) co-infection in HIV-infected adults [6]. However, how published guidelines for clinical management of HBV co-infection reflect treatments received by patients in real-life until now is poorly documented. In particular, it is unclear how often clinician choose guideline-consistent treatments, and what factors may address them to sub-optimal regimens. Noteworthy, disparities in the access to treatment not related to the severity of the disease, but potentially able to counteract any beneficial effects of therapy on CHB hepatitis B have been reported even in industrialized countries [7].

In 2008 the Italian Society of Infectious and Tropical Diseases (SIMIT) conducted a multi-center nationwide survey to evaluate the profiles of patients with CHB cared for by Infectious Diseases Centers (IDCs). In agreement with the Italian National Health Service (NHS), which provides universal access and comprehensive coverage, IDCs are included among the tertiary referral centers allowed to care for patients with CHB. Moreover, Italian NHS stated that only IDCs are allowed to dispense antiretroviral therapy [8].

Therefore, this survey provides an unique opportunity to explore factors, if any, associated with the access to antiviral treatment in a cohort of HIV/HBV co-infected patients cared for in tertiary centers of a developed country where barriers to treatment are theoretically not expected due to comprehensive coverage under NHS. We also analyzed changes over time of the anti-HBV regimens, and the consistency of current regimens with specific European guidelines in force at the time of the study. Finally, we identified factors associated with the receipt of sub-optimal regimens.

## Methods

### Study population and definitions

All of the 110 Italian IDCs were invited by the SIMIT to take part in this cross-sectional survey, and 74 agreed to participate. HIV/HBV co-infected patients were recruited in 41 out of the 74 participating IDCs. Details of the study design and of data collection have been presented elsewhere [9]. Briefly, in each center, all subjects older than 18 years of age with persistent (more than 6 months) HBsAg positivity, and who received care from March 1, 2008 to September 30, 2008 were enrolled in the study. In compliance with the Helsinki Declaration, each enrolled patient signed an informed consent form to abstract and anonymously collect data on their own demographic characteristics, clinical and laboratory values, and treatment history by the referring investigator of each participating centre. A precoded form was used. In accordance with Italian rules, this study was firstly approved by the Ethical Committee of the leading center (Santa Maria Annunziata Hospital, ASL Firenze, Firenze), and then Ethical Committees of all participating centers approved the study protocol.

Chronic hepatitis was diagnosed by liver histology or, for patients without liver biopsy, on the basis of persistent or recurrent abnormal alanine aminotransferase along with HBV DNA greater than 2×10^3^ IU/ml, in the absence of clinical, biochemical, and ultrasound markers of cirrhosis. Cirrhosis was diagnosed by either liver biopsy or the presence of unequivocal clinical, biochemical, and ultrasound signs [10]. The diagnosis of HCC was based on histology, imaging techniques, or a combination of imaging techniques and biochemical parameters [11].

Hepatitis B serum markers (HBsAg, HBeAg and anti-HBe) and anti-HDV antibodies were determined by commercial immunoenzyme assays. Antibodies to HCV were detected by third-generation commercial immunoenzymatic assays. Serum HBV DNA or HIV RNA levels were assessed by polymerase chain reaction amplification.

For the purpose of the present analysis, we included patients tested HIV positive. In order to exclude patients with treatment prescribed but ever started, we considered only anti-HBV treatments taken by patients for at least 1 month. Treatments taken at the time of the survey were considered as current. Patients who restarted the same nucleos(t)ide analogues (NUC) based regimen after a discontinuation of less than one year were considered as treated once.

### EACS treatment guidelines for treatment of HBV co-infected adults

In patients with no indication for anti-HIV therapy (nadir CD4 count >350 cells/mL) EACS guidelines recommended (i) pegylated interferon-α (PEG-IFN); (ii) telbivudine (LdT), adding adefovir (ADV) if HBV DNA is still detectable at week 24; (iii) *de novo* ADV plus LdT; (iiii) early cART initiation including tenofovir (TDF) plus lamivudine/emtricitabine (LAM/FTC). In patients with an indication for anti-HIV therapy (nadir CD4 count ≤350 cells/mL) or already on cART, TDF plus LAM/FTC was recommended. In case of resistance of HBV to LAM, TDF should be added or substituted to one nucleoside reverse transcriptase inhibitor. For cirrhotic patients TDF plus LAM/FTC was the treatment of choice.

For the present analysis, all the above mentioned regimens were considered as guideline consistent (GC); regimens not mentioned in EACS guidelines were classified as unlisted (GU). According to EACS guidelines patients with triple HCV co-infection using interferon-based regimens were considered consistently treated.

### Statistical analysis

Studied population was divided into two groups according to the treatment status at the moment of the survey: never treated patients, and those treated at some time in their life. Groups were compared with respect to a priori-selected demographic and clinical characteristics by using the Pearson *χ*2 test and Wilcoxon test respectively for categorical and continuous variables.

The association of treatment status with selected covariates was studied by using a multivariable logistic model. Results were shown in terms of adjusted Odds Ratios (aOR) with relative 95% Confidence Interval (CI).

Currently treated HIV/HBV co-infected patients were classified according the prescribed antiviral regimen and consistency of regimen with EACS treatment guidelines.

Factors associated with consistency of current treatment with EACS recommendations were studied by using a multivariable logistic model.

To account for the loss in precision and possible bias in the estimates due to missing values in the covariates we were interested in, we used the Multiple Imputation method. Multiple Imputation is based on Missing at Random assumption, stating that the probability of data being missing depends only on the observed data. Consequently, the distribution of the observed data is used to estimate a set of values for the missing data. Multiple data sets are derived and identically analyzed to compute a set of estimates for the parameters of interests. Finally, the estimates are combined according to the Rubin’s rule [12] to obtain the overall estimates, variances and CI’s. Forty imputed datasets were obtained by using the Multiple Imputation by Chained Equations [13,14] approach as implemented in user contributed ice command [15] in Stata [16], then mim command [17] was used to analyze the datasets and combine the estimates.

Within-center correlation was accounted for by computing a robust sandwich estimate of variance-covariance matrix when estimates of imputed data sets were combined.

All tests were two sided and p-values < 0.05 were considered significant.

## Results

The 427 (11.4%) patients classified as HIV/HBV co-infected are considered in the present analysis. Median age at diagnosis was 35 years (interquartile range, 30–42), 75.9% were males, and the majority (83.2%) was born in Italy, 1.6% in East Asia, 9.4% in Sub Saharan Africa and 2.3% in East Europe. A total of 149 (34.9%) patients were HBeAg positive, 78 (18.3%) were HDV co-infected, and 138 (32.3%) HCV co-infected. Seventy-eight (18.3%) patients were alcohol abstinent. Cirrhosis or HCC was present in 37 (8.7%) of the cases. Information on HCV co-infection was missed in 1 (0.2%) patient, HDV co-infection in 27 (6.3%), and alcohol consumption in 139 (32.6%). Three hundred and seventy-four (87.6%) patients have been treated for CHB at some point in their life (Table 1).

**Table 1:** Characteristics of the 427 patients with HIV/HBV co-infection according to anti-HBV treatment status

**Characteristics**	**Never treated patients (n = 53)**	**Treated patients (n = 374)**	**All patients (n = 427)**	**P**
N. enrolled per center, %				
≥20 patients	60.4	52.9	53.9	0.31
Female gender, %	22.6	24.3	24.1	0.79
Immigrant status, %				
foreign birth	18.9	16.8	17.1	0.71
Median age at diagnosis^§^, years				
(IQR)	35 (31–43)	35 (29–42)	35 (30–42)	0.81
Years since HBV diagnosis°, %				<0.001
<2	32.1	7	10.1	
2-10	52.8	59.9	59	
>10	15.1	32.4	30.2	
Alcohol consumption^*^, %				0.22
never	15.1	18.7	18.3	
ever	60.4	47.6	49.2	
not available	24.5	33.7	32.6	
Disease stage, %				
cirrhosis/HCC	3.8	9.4	8.7	0.18
HBeAg, %				0.17
positivity	28.3	35.8	34.9	
not available	1.9	0.3	0.5	
HCV co-infection, %				0.4
yes	24.5	33.4	32.3	
not available	--	0.3	0.2	
HDV co-infection, %				0.07
yes	7.5	19.8	18.3	
not available	9.4	5.9	6.3	

^§^computed on 424 patients; °3 patients not available; * ≥ 20 g/day during at least 6 consecutive months life-time; *IQR*, interquartile range; *HCC*, hepatocellular carcinoma.

Demographic, clinical and virological characteristics were entered in a logistic regression model to identify factors associated with no treatment. The only independent association we observed was with increasing years since HBV diagnosis, which reduced the chance of not being treated (2–10 years: aOR 0.17, 95% CI 0.08-0.37, p <0.001; >10 years: aOR 0.08, 95% CI 0.03-0.26, p <0.001;) (Table 2).

**Table 2:** Logistic regression model in 427 patients with HIV/HBVco-infection according to anti-HBV treatment status

	**Never treated vs treated patients adjusted OR**	
**Characteristics**	**(95% CI)**	**P**
N. enrolled per center^§^		
≥20 patients	1.41 (0.73-2.71)	0.31
Female gender	0.83 (0.39-1.77)	0.64
Immigrant status		
Foreign birth	0.74 (0.3-1.8)	0.51
Age at diagnosis		
10 years increase	0.78 (0.55-1.11)	0.17
Years since diagnosis*		<0.001
2-10	0.17 (0.08-0.36)	
<10	0.08 (0.03-0.25)	
Alcohol consumption		
Ever	1.51 (0.63-3.62)	0.35
Disease stage°		
cirrhosis/HCC	0.39 (0.09-1.78)	0.22
HBeAg		
positivity	0.57 (0.28-1.14)	0.11
HCV co-infection		
yes	0.91 (0.41-2.02)	0.82
HDV co-infection		
yes	0.48 (0.14-1.66)	0.25

Reference: ^§^ = ≤20 patients enrolled; * = <2 years;° = chronic hepatitis. *OR*, odds ratio; *CI*, confidence interval; *HCC*, hepatocellular carcinoma.

Three hundred and thirty-four (89.3%) out of the 374 treated patients were on current anti-HBV treatment, of whom 214 (64.1%) had never been treated before. The most common current anti-HBV regimen was combination therapy of TDF plus LAM/FTC (n = 235, 70.4%), followed by LAM alone (n = 60, 18%), and TDF alone (n = 17, 5.1%). Only 6 (1.8%) patients were on interferon-based treatment, of whom 4 in combination with TDF (Table 3).

**Table 3:** Distribution of antiviral regimens among 334 HIV/HBV co-infected patients on current anti-HBV treatment

**Treatment**	**n**	**(%)**
**TDF + LAM/FTC**	235	70.4
**LAM**	60	18
**TDF**	17	5.1
**Telbivudine**	6	1.8
**PEG-IFN**^**§**^	6	1.8
Other combo NUC^*^	5	1.5
**ETV**	3	0.9
**ADV**	2	0.6
**Total**	334	100

^§^ + TDF = 4; ^*^ADV + LAM =4, ADV + ETV =1.

One hundred and sixty patients (42.8%) had had a history of past treatment; 99 (61.9%) had been previously treated with LAM. At the time of this survey, 70 (68.0%) of these latter patients had been switched to TDF plus LAM/FTC, 10 (9.7%) were using other NUC regimens, only 1 had changed to an interferon-based regimen, and 22 (21.4%) were not being treated. Patients with a previous interferon-based regimen were 36. Twenty (55.6%) were currently on TDF plus LAM/FTC, 7 (19.4%) were on different NUC regimens, 1 patient was retreated, and 8 (22.2%) were not treated. Other NUC-based regimens were previously used by 21 (13.1%) patients, of whom 15 (71.4%) were currently using TDF plus LAM/FTC. Three patients had been switched to TDF plus LAM/FTC, 7 to different NUCs, 1 changed to interferon, and 10 (47.6%) were not being treated.

Two hundred and fifty-five (76.6%) patients were currently treated according to the EACS guidelines for chronic HBV co-infection in force at the time of the survey (Table 4). Patients with an indication for anti-HIV therapy had the highest proportion of GC regimens (79.2%), cirrhotic patients the lowest (65.6%). These figures tended to be statistically significant (p =0.08).

**Table 4:** Distribution of 334 HIV/HBV co-infected patients on current anti-HBV treatment according to EACS treatment guidelines

	**EACS GC regimens**		**EACS GU regimens**	
	**n**	**%**	**n**	**%**
No indication for anti-HIV therapy	30	68.2	14	31.8
Indication for anti-HIV therapy°	202	79.2	53	20.8
Indication for anti-HIV therapy not known^§^	2	--	--	--
Cirrhotic/HCC	21	65.6	11	34.4
Total	255	76.6	78	23.4

*EACS*, European AIDS clinical society; *GC*, guideline consistent; *GU*, guideline unlisted; °according to EACS guidelines for HCV co-infection [6], 1 patient with triple HCV co-infection on PEG-IFN was considered as “consistent”; ^§^3 patients: 2 on TDF plus LAM/FTC considered as “consistent”, 1 not classifiable.

Patients with an indication for anti-HIV therapy and on GC group were 202, 186 (92.1%) were using cART with TDF plus LAM/FTC as backbone. Fifteen (7.4%) others were on cART containing TDF, and 1 patient with triple HCV co-infection was currently treated with PEG-IFN. Comparable figures of cART with TDF plus LAM/FTC were observed either among the 21 cirrhotic patients currently on GC group (100%) or among the 30 patients with no indication for anti-HIV therapy (n = 26, 86.7%). Three (10%) of these latter patients were consistently treated with LdT, and 1 with PEG-IFN. Patients retreated for HBV co-infection and currently on GC group were 86. Sixty-eight (79.1%) had been previously treated with LAM, and 18 with an interferon-based regimen.

Patients falling into GU group were 78 (23.4%). LAM (n = 60, 76.9%) within cART was the most common anti-HBV regimen. Thirty-four (56.7%) of them showed HBV DNA load below 1×10^3^ IU/mL. Both interferon-based regimens and combo NUC were taken by 4 (5.1%) patients. ETV or LdT as mono-therapy were used by 3 patients (3.8%), respectively. Two (2.6%) patients were either on TDF or ADV.

We also analyzed possible factors significantly associated with unlisted anti-HBV regimens. A logistic regression model (Table 5) revealed that a previous anti-HBV treatment and a triple HDV co-infection reduced the chance of GU regimens (aOR 0.4, 95% CI 0.19-0.83, p =0.01; aOR 0.41, 95% CI 0.18-0.93, p =0.03, respectively). Conversely, age at HBV diagnosis and triple HCV co-infection were independently associated with an increased OR of being inconsistently treated (aOR 1.23, 95% CI 1.02-1.48, p =0.03; aOR 2.74, 95% CI 1.39-5.41, p =0.004, respectively).

**Table 5:** Logistic regression model of the odds ratio of the EACS Guidelines Unlisted regimens in 333 HIV/HBV co-infected patients currently on anti-HBV treatment

**Characteristics**	**EACS GU regimens adjusted OR**	
	**(95% CI)**	**P**
N. enrolled per center^§^		
≥20 patients	0.56 (0.23-1.37)	0.2
Female gender	1.92 (0.71-5.18)	0.2
Immigrant status		
Foreign birth	1.4 (0.71-2.74)	0.33
Age at diagnosis		
10 years increase	1.23 (1.02-1.48)	0.03
Years since diagnosis*		0.12
2-10	0.68 (0.24-1.9)	
<10	1.08 (0.39-3.04)	
Alcohol consumption		
Ever	2.21 (0.84-5.86)	0.11
Disease stage°		
Cirrhosis/HCC	1.57 (0.75-3.29)	0.23
HBeAg		
Positivity	0.83 (0.44-1.55)	0.56
HCV co-infection		
Yes	2.74 (1.39-5.41)	0.004
HDV co-infection		
Yes	0.41 (0.18-0.93)	0.03
Previous treatment		
Yes	0.4 (0.19-0.83)	0.01

Reference: ^§^ = ≤20 patients enrolled; * = <2 years;° = chronic hepatitis. *EACS*, European AIDS Clinical Society; *OR*, odds ratio; *CI*, confidence interval; *HCC*, hepatocellular carcinoma.

Finally, we compared HBV DNA and HIV RNA loads, and CD4 cels count of GC patients those of patients on not consistent anti-HBV regimens. Only HBV DNA levels were statistically different (p = 0.03). In particular, proportion of HBV DNA load below median value (2×10^2^ IU/ml) was 66.8% among patients consistently treated and 52.2% among NGC patients.

## Discussion

Features of HIV/HBV co-infected patients enrolled in this cross-sectional study (Table 1) are consistent with those observed in other Italian surveys of co-infected patients cared for by IDCs, such as the proportion of HBeAg-negative cases, and of patients triple co-infected with HCV or HDV [18-20].

Our study shows that in Italy approximately 90% of the HIV/HBV co-infected patients managed by IDCs receive anti-HBV treatment at some point in their life. This figure may be considered remarkably high when taking into account that until a few years ago HIV co-infection had been a factor hampering treatment of viral chronic hepatitis even in industrialized countries with free-drug supply [21,22]. The introduction of TDF plus LAM/FTC, which has also a potent activity against HBV, as an antiretroviral “backbone” largely contributed to increase the proportion of co-infected patients treated for HBV co-infection [23]. This is confirmed by our observation that this NUC combination was the most common regimen used (Table 2).

When demographic, clinical and virological characteristics were entered in a logistic regression model we found that the adjusted ORs of getting HBV antiviral treatment were significantly increased by six times for patients with HBV diagnosis known since 2–10 years (p <0.001) and by more than twelve times for patients with diagnosis known >10 years (p <0.001) (Table 3). Owing to the natural history of chronic hepatitis B, these differences may be considered expected.

The finding that in the field of HIV co-infection the chance to receive anti-HBV therapy is not reduced in potentially disadvantaged populations is intriguing. In fact, inequalities not related to the severity of disease limiting access to therapy are attenuated but not completely eliminated also in those countries with universal coverage [24-26]. Since 1990, in Italy, only IDCs have the duty to dispense comprehensive care for HIV-positive patients with all the costs covered centrally by a government payer. This favored the practice of groups of infectious diseases specialists able to cope with the vast majority of treatment decisions regarding HIV infection and its related conditions. This unique Italian policy of caring for HIV-infected patients played a crucial role either in enhancing the proportion of co-infected patients treated or in reducing significant factors which may limit access to anti-HBV therapy [8].

In our cohort approximately 40% of the treated patients for HBV infection had an history of past treatment, and of these, 75% were on their second/other regimen. Treatment history showed a clear reduction over time of patients using interferon. Reasons may be the high incidence of adverse effects, along with the very low sero-conversion rates of HBeAg or HBsAg observed among HIV/HBV co-infected patients receiving curative regimens as interferon [27,28]. Meanwhile, evidences showed a correlation between prolonged period of cART with dual activity and HBeAg or HBsAg sero-conversion [29,30]. Accordingly, two-thirds of our patients who had been previously on either lamivudine or interferon experienced a switch to TDF plus LAM/FTC. Overall, the proportion of current TDF plus LAM/FTC regimens was twelve times higher than that observed in past anti-HBV treatments.

Our analysis shows that regimens received by 76.6% of HIV/HBV co-infected patients compares favorably with recommendations of EACS guidelines (Table 4). This is despite the fact that co-infected patients present multiple challenges, recommendations regarding the choices of anti-HBV therapy were evolving, and specific EACS guidelines were being published during the enrollment period of this study.

We found that more than two-thirds of patients with no indication for anti-HIV therapy were consistently treated, the overwhelming majority of them having started early cART. As 2008 EACS guidelines were the first to clearly recommend in HBV co-infected patients cART initiation irrespective of CD4+ cells count, it is conceivable that expert clinicians were able, under certain circumstances, to choose regimens later included in guidelines. On the other hand, due to their greater risk of death, proportion of GC treatment should be increased in cirrhotic patients. Interestingly, GC regimens were more likely to reach the goal to significantly decrease HBV DNA load.

Nevertheless, a sizeable minority of patients was on a suboptimal anti-HBV treatment, the majority (n =60, 77%) on LAM alone. The finding that more than half on them showed low levels of HBV DNA load suggests that in some patients clinicians seemed to change a regimen, which involves also HIV suppression, only if these measurements started to deteriorate.

Due to the predominance of intravenous drug users among HIV/HCV co-infected patients in Italy [31], it is conceivable that high rates of drug addiction among these patients may contribute to increase the rate of suboptimal treatments. Our findings that HCV co-infection increased of more two times (p = 0.004) the chance of falling into the GU group (Table 5), with triple co-infected patients representing approximately half of those on not consistent LAM stress the issue of adherence. Unfortunately, information on route of infection was not asked for. Conversely, our observation that the chance of sub-optimal treatment was significantly lower (p = 0.01) in patients on second line anti-HBV regimen confirms the practice of switching towards optimal regimens when available made by IDC clinicians. Patients with triple HDV co-infection, known to be at an increased risk of an unfavorable outcome, also had a decreased risk (p = 0.03) to be classified into GU group.

This study has limitations, mainly deriving from the design of the study. Our cohort was not randomly sampled, and recruitment of the IDCs was voluntary-based. Therefore, we cannot rule out the possibility that enrolled patients was not representative of the entire population of co-infected patients cared for by the participating centres. Nevertheless, the proportion of patients who denied the anonymous collection of medical data from their charts was very small, and the study was conducted in those IDCs where the majority of HIV-infected individuals are cared for [32].

We were unable to investigate the reasons for and the period of treatment change. No information was available regarding socio-economic, or educational distribution of the cohort. Nor did we had information on the reasons for not receiving antiviral treatment, such as patient refusal.

Using Multiple Imputation, we were able to exploit all the information available and avoid the drawbacks (loss in precision and potential bias of the estimates) related to the exclusion from the analysis of the patients who had missing information on any of the predictors. Extrapolations of our results to clinical settings of other developed countries must be carried out with caution.

It is also possible that patients could have received treatment at outside facilities. However, the high proportion of treated patients by participating centres makes likelihood for the receipt of treatment at other settings very low.

## Conclusions

Although further long-term studies need to be performed to confirm our observations, results of our analysis have practical implications. A health system aimed at providing free of charge and comprehensive care for HIV-infected individuals by groups of specialists allows to dispense anti-HBV therapy to a very high proportion of co-infected patients in absence of disparities which limit access to treatment. In order to slow progression of HBV disease in co-infected patients, expert physicians understand the need to quickly implement specific guidelines, and in some cases even to anticipate them. Nevertheless, our study identified scenarios in which clinical practice deviates from updated recommendations, as in case of low adherence or sub-optimal regimens with effective anti-HBV response.

## Abbreviations

ADV: Adefovir; aOR: Adjusted odds ratio; cART: Combined antiretroviral therapy; CHB: Chronic hepatitis B; CI: Confidence interval; EACS: European AIDS clinical society; FTC: Emtricitabine; GC: Guideline consistent; GU: Guideline unlisted; HBV: Hepatitis B virus; HCC: Hepatocellular carcinoma; HCV: Hepatitis C virus; HIV: Human immunodeficiency virus; LAM: Lamivudine; LdT: Telbivudine; NHS: National health service; NUC: Nucleos(t)ide analogues; PEG-IFN: Pegylated interferon-α; SIMIT: Italian society of infectious & tropical diseases; TDF: Tenofovir.

## Participants to the coorte epatiti B SIMIT/COESI-B (HIV) group

Giampiero Carosi, A.O. Spedali Civili di Brescia, Istituto di Malattie Infettive e Tropicali, Brescia; Francesco Mazzotta, Ospedale S.M. Annunziata, Malattie Infettive, Antella; Giorgio Antonucci, INMI Lazzaro Spallanzani, Roma; Francesco Leoncini A.O.U. Careggi, U.O. di Malattie Infettive, Firenze; Evangelista Sagnelli, A.O. S. Sebastiano Caserta, Malattie Infettive, Caserta; Giovanni Cassola, E.O. Ospedali Galliera, Malattie Infettive, Genova; Paolo Antonio Grossi, Univ. degli Studi dell’Insubria, Malattie Infettive, Varese; Vincenzo Guadagnino, Ospedali Civili Riuniti G. Rummo, Malattie Infettive, Benevento; Gabriella Pagano, Az. Osp. San Martino, U.O. Malattie Infettive, Genova; Giuseppe Foti, A.O. Bianchi-Melacrino-Morelli, U.O. Malattie Infettive, Reggio Calabria; Giorgio Scalise, Ospedale di Torrette, Malattie Infettive, Ancona; Gabriella Verucchi, Università degli Studi di Bologna - Malattie Infettive, Bologna; Massimo Galli, Ospedale Luigi Sacco, Istituto Malattie infettive, Milano; Franco Marranconi, ULSS 4 Malattie Infettive, Schio; Andrea Gori, A.O. San Gerardo di Monza, Malattie Infettive, Monza; Massimo Andreoni, Ospedale degli Infermi, Malattie Infettive, Biella; Stefania Artioli, ASL 5 Regione Liguria, Malattie Infettive, La Spezia; Arlotti, ASL Rimini, Malattie Infettive, Rimini; Roberto Rinaldi A.O. Padova, Malattie Infettive, Padova; Giulio De Stefano, Ospedale Madonna delle Grazie, U.O. Malattie Infettive, Matera; Giustino Parruti, Asl Pescara, UOC Malattie Infettive, Pescara; Sauro Luchi, Ospedale di Lucca, Malattie Infettive, Lucca; Gianpaolo Natalini Raponi, Ospedale Provinciale, U.O. AIDS, Rieti; Lucina Titone, Università di Palermo, Malattie Infettive, Palermo; Nicola Abrescia, A.O. Domenico Cotugno, Malattie Infettive, Napoli; Anna Orani, Ospedale A. Manzoni, Malattie Infettive, Lecco; Francesco Alberici, AUSL Piacenza, Malattie Infettive, Piacenza; Roberto Luzzati, Ospedale Maggiore A.O. Trieste, Malattie Infettive, Trieste; Guido Raineri, A.O. S. Croce e Carle, Malattie Infettive e Tropicali, Cuneo; Orlando Armignacco, ASL Viterbo, Malattie Infettive, Viterbo; Nicola Petrosillo, INMI Lazzaro Spallanzani, Malattie Infettive, Roma; Franco Baldelli, Università di Perugia, Malattie Infettive, Perugia; Marco Dini, Ospedali Riuniti, Malattie Infettive, Ancona; Gaetano Filice, IRCSS Fondazione San Matteo, Malattie Infettive e Tropicali, Pavia; Carla Cellesi, Policlinico Le Scotte, Malattie Infettive, Siena; Maria Stella Mura, Università di Sassari, Malattie Infettive, Sassari.

## Competing interests

The authors declare that they have no competing interests.

## Authors’ contributions

GA: protocol design, overall study design, data review, interpretation of results, manuscript preparation and review; FM: conceived the study, protocol design, supervision and management of study implementation, manuscript review; CA: data analysis, manuscript review; EG: study design, interpretation of results, manuscript review; MP: study advice, enrolled study patients and reviewed the manuscript; GDS: enrolled study patients and reviewed the manuscript; PG: enrolled study patients and reviewed the manuscript; NP: enrolled study patients and reviewed the manuscript; GP: enrolled study patients and reviewed the manuscript; GC: enrolled study patients and reviewed the manuscript; AO: enrolled study patients and reviewed the manuscript; CS: enrolled study patients, manuscript preparation and review; OA: protocol design, study design, manuscript preparation and review; ES: protocol design, study design, manuscript preparation and review. All authors read and approved the final manuscript.
